# *Acacia senegal* gum attenuates systemic toxicity in CCl_4_-intoxicated rats via regulation of the ROS/NF-κB signaling pathway

**DOI:** 10.1038/s41598-021-99953-y

**Published:** 2021-10-13

**Authors:** Marwa M. Abu-Serie, Asmaa F. Hamouda, Noha H. Habashy

**Affiliations:** 1 Department of Medical Biotechnology, Genetic Engineering, and Biotechnology Research Institute, City of Scientific Research and Technological Applications (SRTA-City), New Borg EL-Arab, 21934 Alexandria Egypt; 2 Biochemistry Department, Faculty of Science, Alexandria University, Alexandria, 21511 Egypt

**Keywords:** Interleukins, Transforming growth factor beta, Biochemical assays, Liquid chromatography, Rat

## Abstract

*Acacia senegal* (AS) gum (Gum Arabic) is a natural emulsifier exudate from the branches and trunk of Acacia trees and it is recognized by the Food and Drug Administration (FDA) agency as a secure dietary fiber. The present research evaluated the systemic oxidative and necroinflammatory stress induced by CCl_4_ administration and the alleviating effect of AS gum aqueous extract (ASE, 7.5 g/Kg b.w.). The results demonstrated the presence of certain phenolic compounds in ASE, as well as its in vitro potent scavenging ability against ABTS (2,2′-azino-bis(3-ethylbenzothiazoline-6-sulfonic acid), NO, and lipid peroxide radicals. Also, the outcomes revealed an improvement in the CCl_4_-induced liver, lung, brain, and spleen toxicity by reducing the levels of ROS, lipid peroxidation, NO, and the gene expression of NF-κB and its relevant ROS-mediated inflammatory genes. In contrast, the total antioxidant capacity (TAC), as well as the enzymatic and non-enzymatic antioxidants, were significantly upregulated in these organs after the treatment with ASE. These results were confirmed by improving the morphological features of each organ. Therefore, ASE can ameliorate the systemic toxicity caused by CCl_4_ via regulation of the ROS/NF-κB signaling pathway in the rat organs, which is owed to its phytochemical composition.

## Introduction

Oxidative stress exhibits an asymmetry between the exposure to reactive oxygen species (ROS) and the ability of the physiological system to directly scavenge the reactive intermediates or adjust the outcome damage^1^. Disturbances in the normal redox state of cells can produce lethal results by building ROS that damage all cell parts, involving lipids, proteins, and DNA^1,2^. ROS plays also a crucial role in the pathogenesis of various human organs, including the liver, heart, lung, spleen, and kidney^3,4^. ROS has an important role in the modulation of the nuclear factor-kappa (NF-κ)B pathway, so there is an interplay between this pathway and cellular oxidative stress. NF-κB is a member of a family of inducible transcription factors involved in immunological and inflammatory responses induced by pathogens or cytokines, along with cell proliferation and survival. It is a key player in the stimulation of pro-inflammatory genes in both innate and adaptive immune cells, including inducible nitric oxide synthase (iNOS), cyclooxygenase (COX)-2, and tumor necrosis factor (TNF)-α^5^. Various toxic chemicals, such as carbon tetrachloride (CC1_4_), bromobenzene, aromatic hydrocarbons, and methanol^6^, are implicated in the development of organ damage by modulating the ROS/NF-κB signaling pathway^6,7^. The CCl_4_ is a prevalent hepatotoxin used to provoke liver complications in various experimental studies^8,9^. Its hepatotoxic effect has been histologically demonstrated to cause fibrosis, hepatocellular death, and cirrhosis^10,11^. The CCl_4_ is incorporated with its quick break by cytochrome P450 (CYP2E1) in liver cells, providing trichloromethyl radicals (CCl_3_*) driving lipid peroxidation and subsequent membrane impairment^4,10^. In addition to this, CCl_4_ caused activation of hepatic Kupffer cells that generate inflammatory mediators and ROS, yielding an ending in hepatic parenchymal cells' distress^2,11^. Other organs such as the kidney, lung, and spleen can also be harmed by CCl_4_^12^.

Nowadays, numerous functional foods and their ingredients are involved in the amelioration of the chemical's toxicity due to their antioxidant and anti-inflammatory roles^13–17,18^. *Acacia Senegal* (AS) gum (Gum Arabic), which is a naturally occurring exudate derived from the trunk and branches of AS and *Acacia seyal* trees, is one of these functional foods that have numerous medicinal effects. It is a dietary fibre that is neutral or slightly acidic and occurs naturally as a mixed magnesium, calcium, and potassium salt polysaccharidic acid^19,20^. AS gum has anti-inflammatory and antioxidant activities and protects from kidney, liver, and heart injury. It also ameliorates some biochemical, physiological, and behavioral effects in rats^21,22^ besides its ability to relieve the disadvantageous effects of chronic kidney malfunction in humans^20,22^. Few previous studies have assessed the ameliorative effect of AS gum on the drug and chemical-induced toxicity^23,24^, but no study has evaluated its effect on CCl_4_-induced systemic damage. Thus, the present work was conducted to evaluate the therapeutic effect of AS gum aqueous extract (ASE) on the CCl_4_-induced toxicity in rat liver, lung, brain, and spleen tissues. The necroinflammatory and oxidative stress mediators have been examined due to their critical role in systemic destruction. Moreover, the phytochemistry and the in vitro antioxidant activity of ASE were assessed to provide more persuasive proof of its therapeutic opportunities.

## Results

### Phenolic content and antioxidant activity of ASE

**Figure 1:**
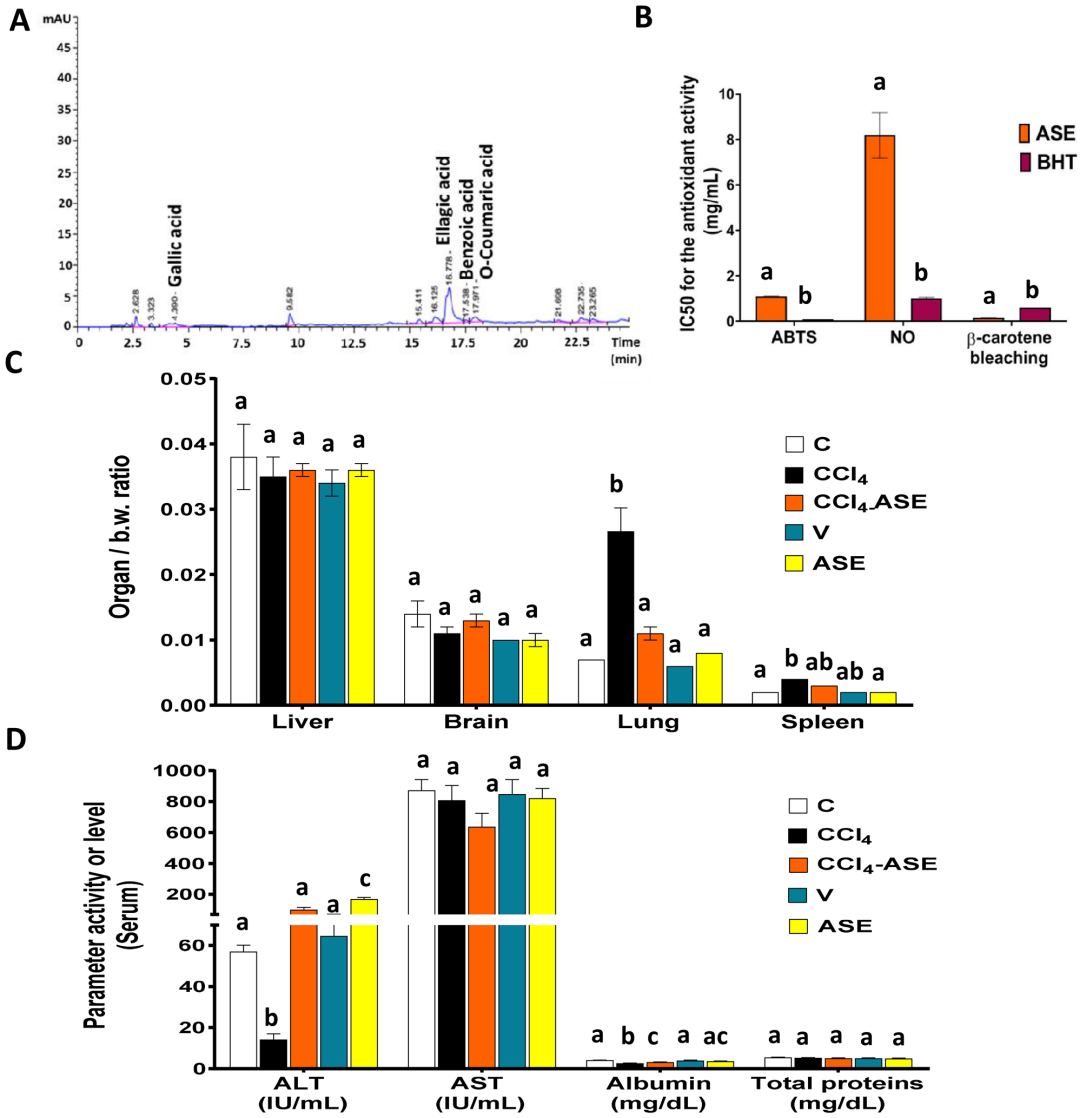
In vitro characterization of ASE, organ weights, and serum analysis of rats in all the studied groups. (**A**) HPLC analysis of the ASE. (**B**) in vitro antioxidant activities of ASE using three different antioxidant systems (**C**) organ weight/body weight (b.w.) ratio. (**D**) liver function parameters in the serum of rats. Results are expressed as mean ± S.E of 7 animals. *C* control untreated rats, *V* olive oil (vehicle of CCl_4_)-administered rats (0.5 ml/kg b.w., ip, 6 times), *CCl*_*4*_ rats with systemic toxicity induced by CCl_4_ injection (1 mL/kg b.w., ip, 6 times); *CCl*_*4*_*-ASE*, rats with systemic toxicity after their oral treatment with ASE (7.5 g/kg b.w.) for 10 days. *ASE*, normal rats were administered only ASE (7.5 g/kg b.w.) for 10 days. Different letters refer to the significance at *P* < 0.05; CCl_4_-ASE group was compared with the CCl_4_ group, while V and ASE groups were individually compared with the C group. *ABTS* 2,2′-azino-bis (3-ethylbenzothiazoline-6-sulfonic acid), *ALT* alanine aminotransferase, *AST* aspartate aminotransferase, *BHT* butylated hydroxytoluene, *IC50* 50% inhibitory concentration, *NO* nitric oxide radical.

The phytochemical analysis showed that ASE contains some amounts of phenolic compounds (0.717 ± 0.102 mg gallic acid eq/g ASE) and has no flavonoids. Certain types of these phenolic compounds were identified by HPLC (Fig. 1A), such as gallic acid (0.1 µg/g ASE), ellagic acid (1.6 µg/g ASE), benzoic acid (0.3 µg/g ASE), and O-coumaric acid (0.1 µg/g ASE). However, the retention times (RT) of the other tested standard phenolics (catechol, caffeine, vanillin, vanillic acid, caffeic acid, syringic acid, p-coumaric acid, cinnamic acid, and salicylic acid) didn’t match any RT in the ASE chromatogram.

Regarding the antioxidant activity of ASE (Fig. 1B), it exhibited potent anti-lipid peroxidation activity (β-carotene bleaching activity) that was significantly (*P* < 0.05) higher than that of butylated hydroxytoluene (BHT) by 73.754%. In addition, it showed high scavenging ability against the ABTS (2,2'-azino-bis(3-ethylbenzothiazoline-6-sulfonic acid) cation radical and NO radical, but less than that of BHT by 1187.058% and 714.527%, respectively.

### The ameliorating effect of ASE on CCl_4_-induced systemic toxicity

The potency of ASE against CCl_4_-induced toxicity in the liver, lung, brain, and spleen was evaluated by its ability to restore the cellular redox state and reduce necroinflammation in these tissues.

### Organ/body weight ratio and liver function markers

The organ/b.w ratio results showed a non-significant difference between all the studied groups for the liver and brain tissues. However, a significant (*P* < 0.05) elevation in these ratios was observed in the lung and spleen tissues after CCl_4_ administration only, by 276.431 and 86.067%, respectively (Fig. 1C).

The serum analysis for the liver function markers (Fig. 1D) showed a significant (*P* < 0.05) decline in the ALT activity (75.146%) and albumin level (36.707%) in the rats that were injected with CCl_4_ compared to the control rats. The remaining studied serum markers (AST and total proteins) revealed slight changes (7.323% and 3.287%, respectively) from the control. Concerning rats in the CCl_4_-ASE group, the ALT activity and albumin level were significantly (*P* < 0.05) increased by 604.471% and 21.081%, respectively, but there was no change in the AST activity (21.254%) and total protein level (6.134%) compared to those in the CCl_4_ group. The V group displayed a non-significant difference in the ALT activity (13.255%), AST activity (2.834%), albumin level (3.584%), and total protein level (7.658%), relative to the control. Similarly, the serum of rats that were administered ASE alone showed slight differences in the AST activity (5.883%), albumin level (12.640%), and total protein level (9.550%), relative to control. In contrast, the activity of ALT was significantly (*P* < 0.05) elevated (195.865%) in the rats of this group compared to that in the C group.

### Improving the CCl_4_-induced hepatic redox state disturbance by ASE

**Figure 2:**
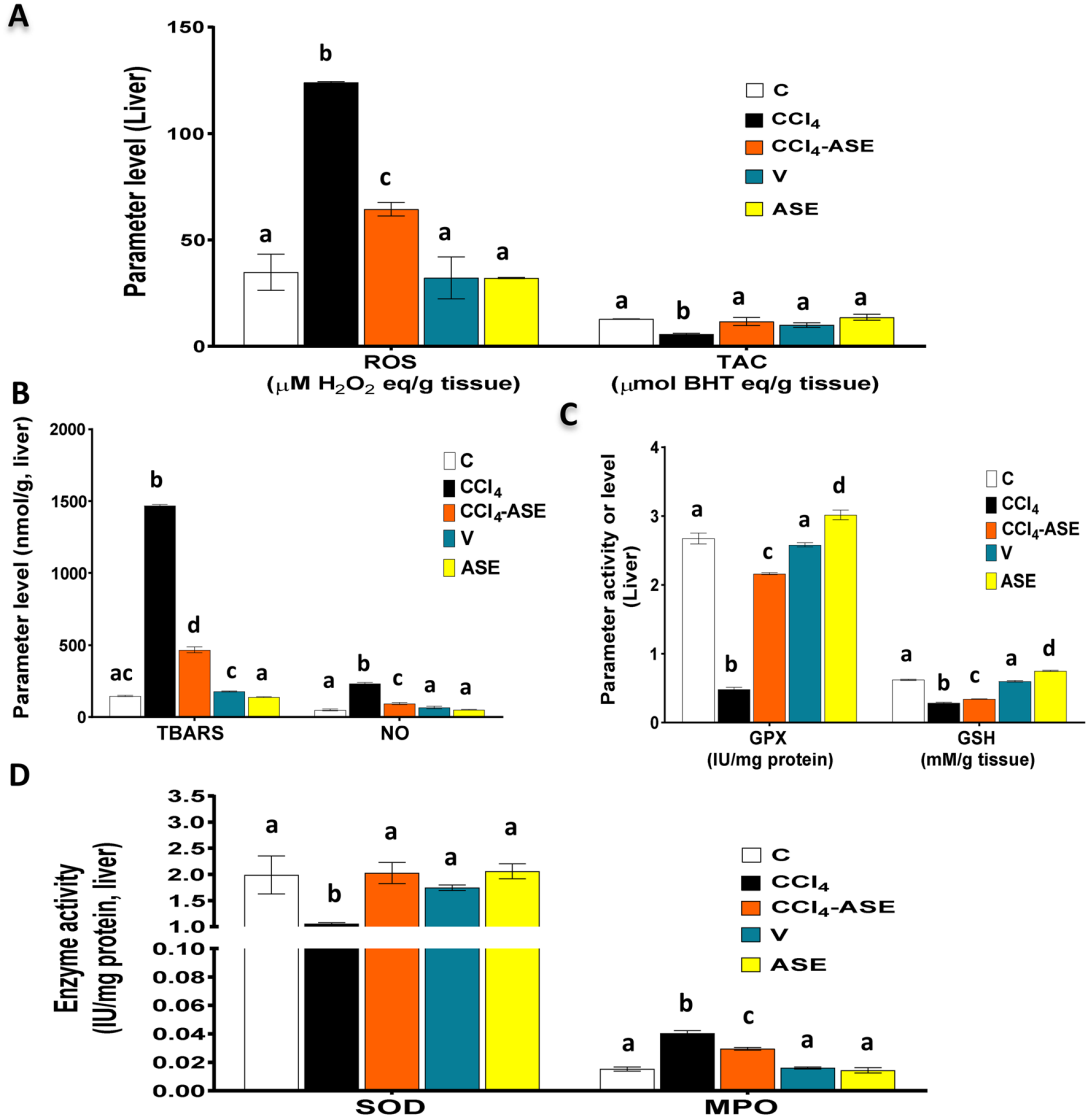
Ameliorating effect of ASE on the CCl_4_-induced hepatic redox state disturbance. (**A**) reactive oxygen species (ROS) and total antioxidant capacity (TAC) levels. (**B**) thiobarbituric acid reactive substances (TBARS) and nitric oxide (NO) levels. (**C**) reduced glutathione (GSH) level and glutathione peroxidase (GPX) activity. (**D**) superoxide dismutase (SOD) and myeloperoxidase (MPO) activities. Results are expressed as mean ± S.E of 7 animals. *BHT* butylated hydroxytoluene, *C* control untreated rats, *V* olive oil (vehicle of CCl_4_)-administered rats (0.5 ml/kg b.w., ip, 6 times), *CCl*_*4*_ rats with systemic toxicity induced by CCl_4_ injection (1 mL/kg b.w., ip, 6 times), *CCl*_*4*_*-ASE* rats with systemic toxicity after their oral treatment with ASE (7.5 g/kg b.w.) for 10 days. *ASE* normal rats were administered only ASE (7.5 g/kg b.w.) for 10 days. Different letters refer to the significance at *P* < 0.05; CCl_4_-ASE group was compared with the CCl_4_ group, while V and ASE groups were individually compared with the C group.

Figure 2A,B, and D show that CCl_4_ induced hepatic oxidative stress, which was explained by a significant (*P* < 0.05) elevation of ROS (256.163%), NO (369.80%), and lipid peroxidation (904.382%) levels, as well as myeloperoxidase (MPO) activity (163.715%) compared to the C group. This was connected with a significant (*P* < 0.05) decrease in the TAC (55.495%), enzymatic [superoxide dismutase "SOD" (46.893%) and glutathione peroxidase "GPX" (81.913%)] and non-enzymatic [reduced glutathione "GSH" (54.123%)] antioxidants relative to the C group (Fig. 2A,C,D). While the administration of ASE after CCl_4_ injection (CCl_4_-ASE group) significantly (*P* < 0.05) reduced the levels of ROS, NO, TBARS, and the activity of MPO by 48.057%, 59.599%, 68.208%, and 26.847%, respectively, relative to the CCl_4_ group (Fig. 2A,B,D). Moreover, ASE significantly (*P* < 0.05) augmented the levels of hepatic TAC (103.374%) and GSH (19.864%), and the activities of GPX (346.491%) and SOD (92.01%) compared to those in the CCl_4_ group (Fig. 2A,C,D).

Regarding rats that were injected with olive oil alone (V group), they showed non-significant changes in all the studied oxidative stress parameters. The changes in ROS, TAC, TBARS, NO, GPX, GSH, SOD and MPO were 7.520%, 21.979%, 21.823%, 34.118%, 3.536%, 3.339%, 12.207%, and 4.219%, respectively, compared to those in the control rats. On the other hand, the rats that were administered ASE alone for 10 days (ASE group) showed a non-significant change in the levels of ROS, TAC, TBARS, and NO and the activity of SOD and MPO compared to the control (7.779%, 6.143%, 5.492%, 1.859%, 3.550%, and 5.953%, respectively). While the activity of GPX and the level of GSH were significantly (*P* < 0.05) elevated by 12.783% and 20.611%, respectively, when compared to those in the C group (Fig. 2C).

### Improving the CCl_4_-induced brain redox state disturbance by ASE

**Figure 3:**
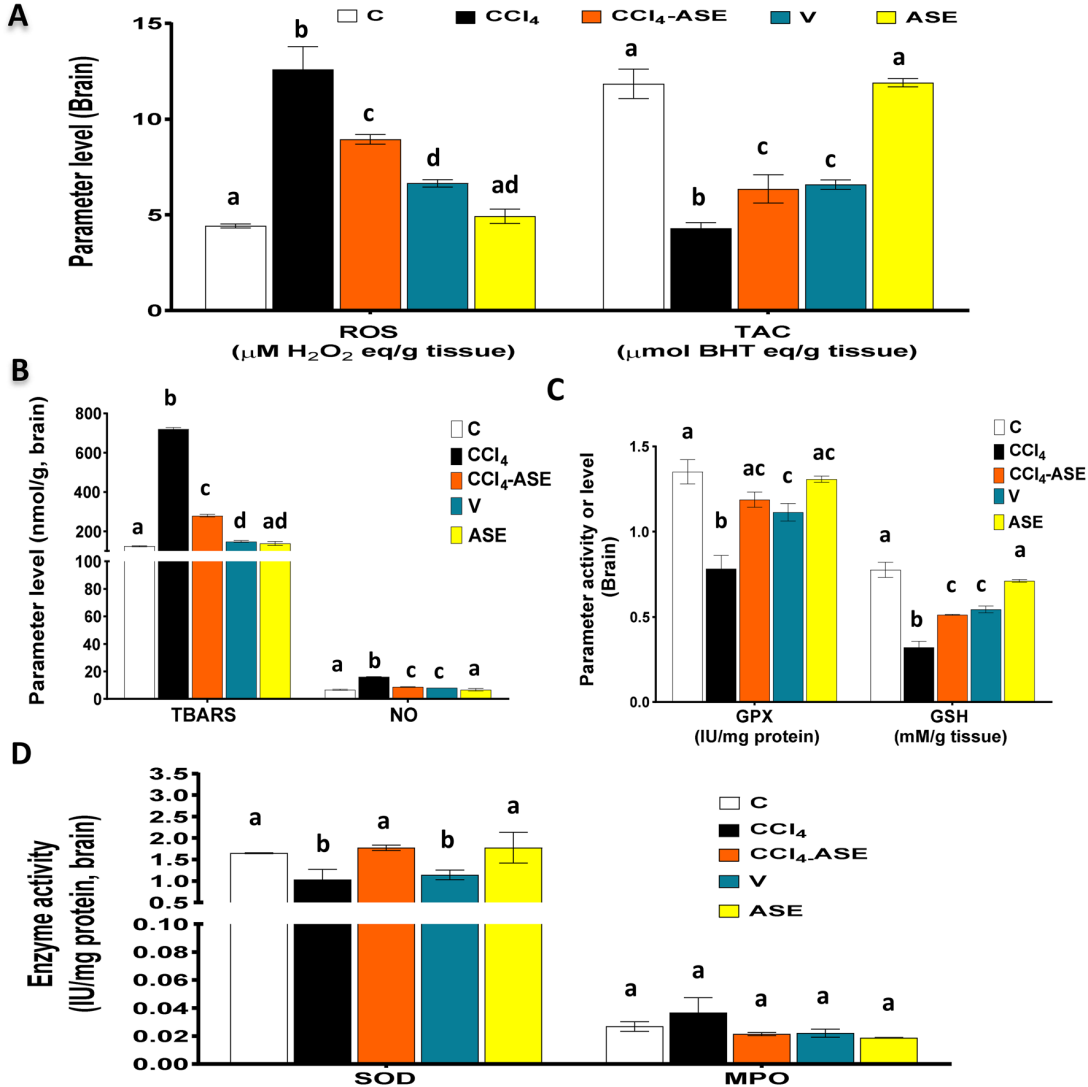
Ameliorating effect of ASE on the CCl_4_-induced redox state disturbance in the brain tissue. (**A**) reactive oxygen species (ROS) and total antioxidant capacity (TAC) levels. (**B**) thiobarbituric acid reactive substances (TBARS) and nitric oxide (NO) levels. (**C**) reduced glutathione (GSH) level and glutathione peroxidase (GPX) activity. (**D**) superoxide dismutase (SOD) and myeloperoxidase (MPO) activities. Results are expressed as mean ± S.E of 7 animals. *BHT* butylated hydroxytoluene, *C* control untreated rats, *V* olive oil (vehicle of CCl_4_)-administered rats (0.5 ml/kg b.w., ip, 6 times), *CCl*_*4*_ rats with systemic toxicity induced by CCl_4_ injection (1 mL/kg b.w., ip, 6 times), *CCl*_*4*_*-ASE* rats with systemic toxicity after their oral treatment with ASE (7.5 g/kg b.w.) for 10 days. *ASE*, normal rats were administered only ASE (7.5 g/kg b.w.) for 10 days. Different letters refer to the significance at *P* < 0.05, CCl_4_-ASE group was compared with the CCl_4_ group, while V and ASE groups were individually compared with the C group.

Figure 3A and B show the toxic effect of CCl_4_ on brain tissue through the induction of oxidative stress, which was indicated by the significant (*P* < 0.05) rise in the levels of intracellular ROS (185.182%), NO (140.579%), and TBARS (480.468%) as compared to the C group. Otherwise, there was a non-significant (36.179%) difference in the MPO activity in the brain tissue of the CCl_4_-injected rats and the control ones (Fig. 3D). Moreover, the cellular redox state (TAC), the activity of GPX and SOD, and the level of GSH were extremely reduced by 63.778%, 42.000%, 37.463%, and 58.677%, respectively, compared to the C group (Fig. 3A,C,D). Treatment of CCl_4_-injected rats with ASE (CCl_4_-ASE group) significantly incremented the cellular redox state of the brain tissue. This was shown by a significant (*P* < 0.05) decrease in the levels of accumulated intracellular ROS (28.951%), TBARS (61.184%), and NO (46.038%), respectively relative to that of the CCl_4_ group (Fig. 3A,B). In contrast, the data analysis found a significant increase (*P* < 0.05) in the TAC (48.006%) and GSH (59.799%) levels and the GPX (51.519%) and SOD (72.019%) activities after ASE treatment compared to those in the CCl_4_ group (Fig. 3A,C,D). While a non-significant change (41.511%) was observed in the activity of MPO in this group compared to those in the CCl_4_ group (Fig. 3D).

The results also detected a significant (*P* < 0.05) elevation in the ROS, TBARS, and NO levels in the brain of rats injected with olive oil only (V group) compared to those in the control group (Fig. 3A,B). However, the antioxidant indices in the brain of these rats (Fig. 3A,C,D), such as the TAC (44.444%), GSH (29.822%), GPX (17.578%), and SOD (30.741%) were significantly (*P* < 0.05) diminished relative to control. But the MPO activity was decreased insignificantly (17.903%) in the brain tissue of these rats, relative to the control group (Fig. 3D).On the other hand, administration of ASE alone (ASE group) had insignificant effects on the levels of ROS (11.584%), TAC (0.500%), TBARS (11.519%), NO (0%), and GSH (8.239%) or the activities of GPX (3.209%), SOD (7.587%), and MPO (30.220%) compared to those in the control group.

### Improving the CCl_4_-induced lung redox state disturbance by ASE

**Figure 4:**
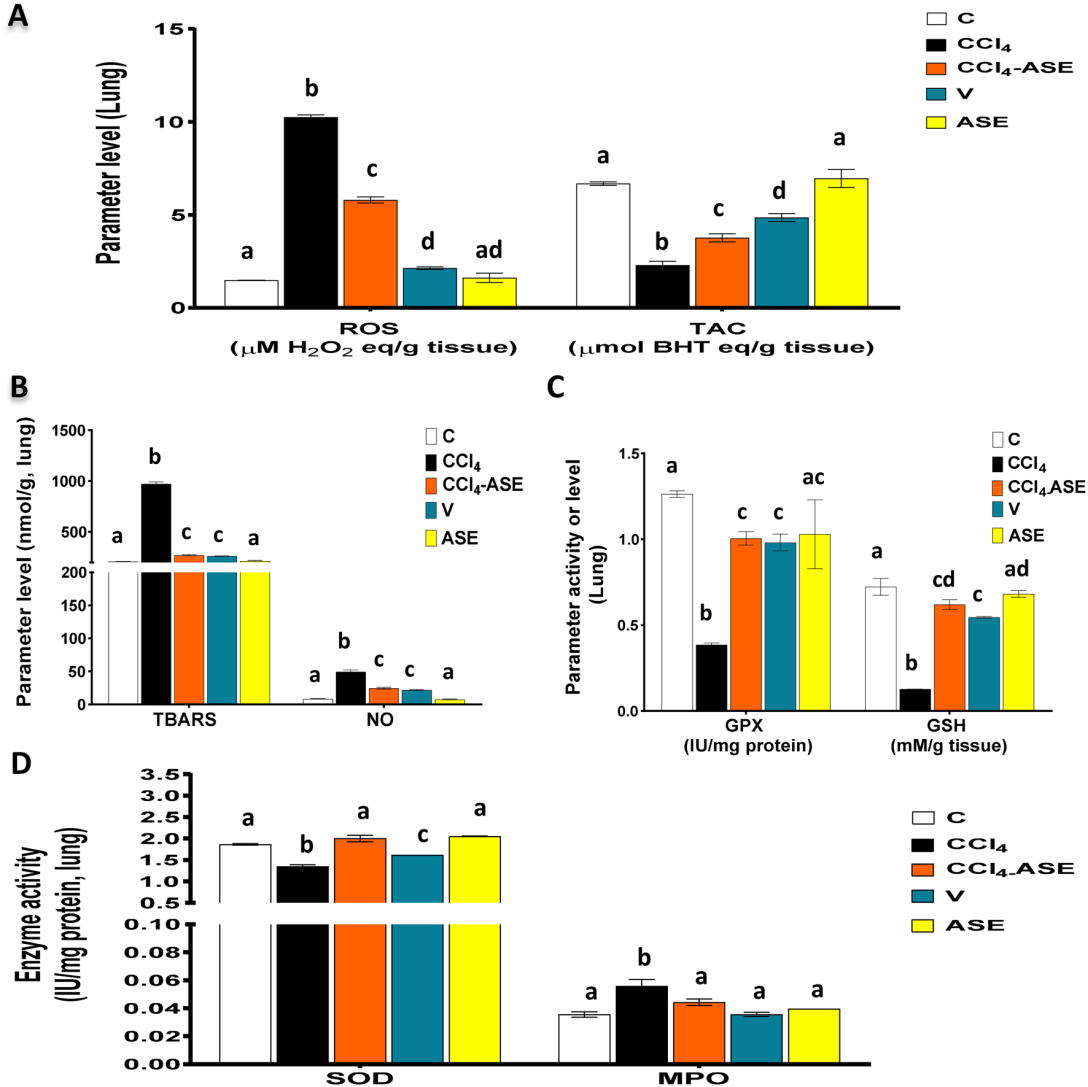
Ameliorating effect of ASE on the CCl_4_-induced redox state disturbance in the lung tissue. (**A**) reactive oxygen species (ROS) and total antioxidant capacity (TAC) levels. (**B**) thiobarbituric acid reactive substances (TBARS) and nitric oxide (NO) levels. (**C**) reduced glutathione (GSH) level and glutathione peroxidase (GPX) activity. (**D**) superoxide dismutase (SOD) and myeloperoxidase (MPO) activities. Results are expressed as mean ± S.E of 7 animals. *BHT* butylated hydroxytoluene, *C* control untreated rats, *V* olive oil (vehicle of CCl_4_)-administered rats (0.5 ml/kg b.w., ip, 6 times), *CCl*_*4*_ rats with systemic toxicity induced by CCl_4_ injection (1 mL/kg b.w., ip, 6 times), *CCl*_*4*_*-ASE* rats with systemic toxicity after their oral treatment with ASE (7.5 g/kg b.w.) for 10 days. *ASE*, normal rats were administered only ASE (7.5 g/kg b.w.) for 10 days. Different letters refer to the significance at *P* < 0.05, CCl_4_-ASE group was compared with the CCl_4_ group, while V and ASE groups were individually compared with the C group.

Figure 4A and B show that CCl_4_ caused harm to the lung redox state with a massive rise in the levels of ROS (591.297%) and NO (483.908%), compared to those in the C group, which led to an incredible formation of TBARS (361.120%). Also, compared to that of the control group, there was a significant increase in the MPO activity by 57.318%. Conversely, the lung enzymatic (GPX and SOD) and non-enzymatic (GSH) antioxidants were enormously reduced by 69.426%, 27.635%, and 82.387%, respectively, compared to those in the C group (Fig. 4C,D). Consequently, TAC of lung tissue was significantly dropped in the CCl_4_-injected rats by 65.780%, related to the control group (Fig. 4A). The treatment with ASE after CCl_4_-injection (CCl_4_-ASE group) improved the redox state in lung tissue extremely. This was attributed to the significant (*P* < 0.05) decrease in the studied oxidative stress parameters, ROS, NO, TBARS, and MPO levels, by 43.448%, 50.098%, 72.211%, 20.594%, respectively, compared to those in the CCl_4_ group (Fig. 4A,B,D). Besides, ASE significantly (*P* < 0.05) enhanced the lung antioxidant system (GPX, GSH, and SOD) by 160.157%, 386.431%, and 48.505%, relative to the CCl_4_ group (Fig. 4C,D). These results led to an increase in the lung tissue TAC (64.683%) related to that in the CCl_4_ group (Fig. 4A).

On the other hand, the lung tissue of rats in the vehicle (V) group showed an alteration in the redox state parameters. Hence, ROS, NO, and TBARS were significantly (*P* < 0.05) increased by 43.619%, 158.818%, and 24.490%, compared to the C group. Also, the antioxidant parameters, including GPX (22.390%), GSH (24.611%), and SOD (3.469%) were depleted significantly (*P* < 0.05), and as a result, the TAC (27.389%) was decreased compared to the control group (Fig. 4A,C,D), while the activity of MPO in the lung of the rats in this group was insignificantly increased (0.456%), compared to that in the C group. Nevertheless, the administration of ASE alone for 10 days (ASE group) displayed non-significant changes in the studied oxidative stress parameters in lung tissue compared to the C group. These parameters include ROS (12.146%), TBARS (2.306%), NO (10.344%), MPO (11.409%), GPX (18.502%), GSH (5.813%), SOD (10.135%), and TAC (4.137%).

### Improving the CCl_4_-induced spleen redox state disturbance by ASE

**Figure 5:**
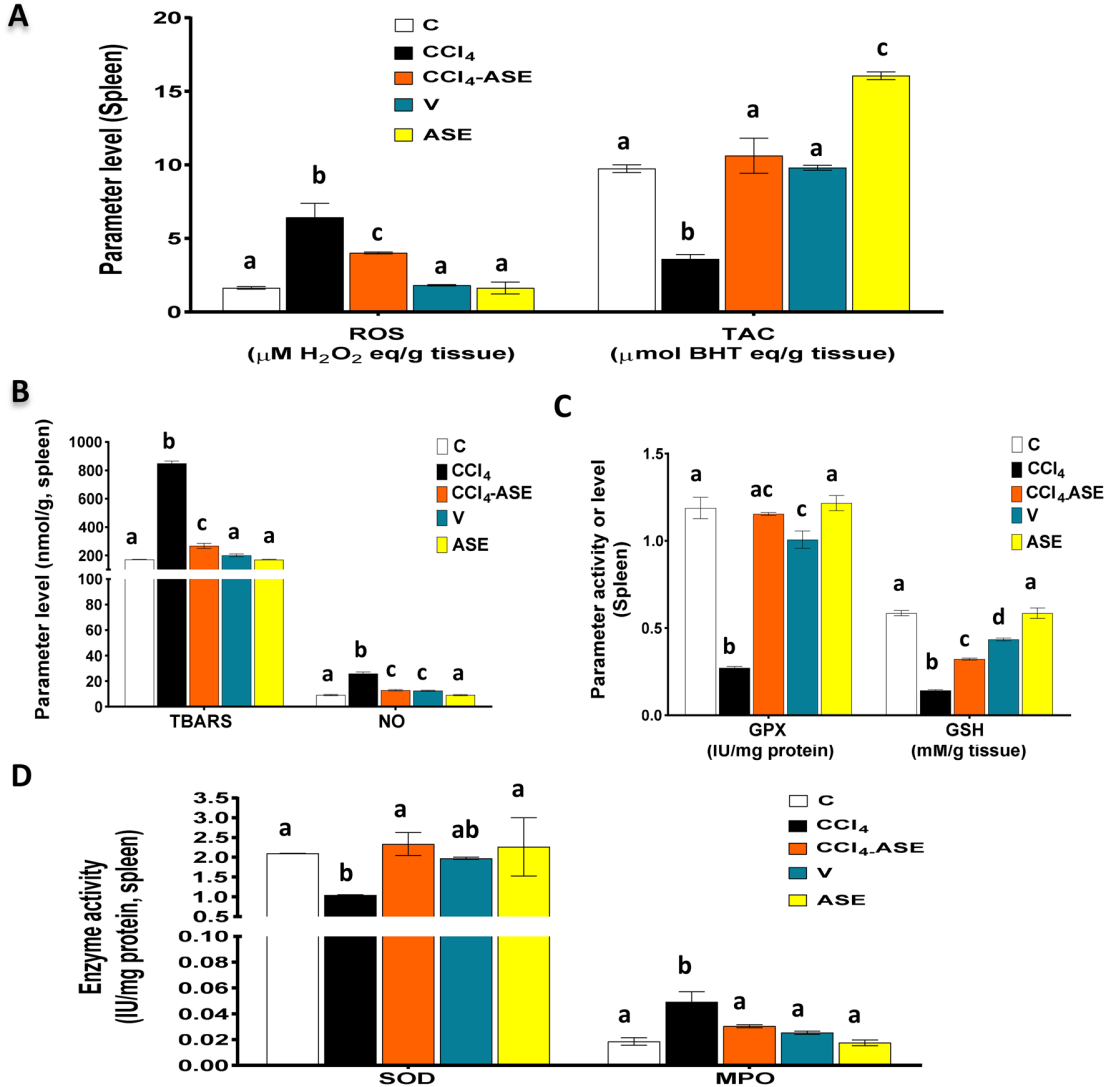
Ameliorating effect of ASE on the CCl_4_-induced redox state disturbance in the spleen tissue. (**A**) reactive oxygen species (ROS) and total antioxidant capacity (TAC) levels. (**B**) thiobarbituric acid reactive substances (TBARS) and nitric oxide (NO) levels. (**C**) reduced glutathione (GSH) level and glutathione peroxidase (GPX) activity. (**D**) superoxide dismutase (SOD) and myeloperoxidase (MPO) activities. Results are expressed as mean ± S.E of 7 animals. *BHT* butylated hydroxytoluene, *C* control untreated rats, *V* olive oil (vehicle of CCl_4_)-administered rats (0.5 ml/kg b.w., ip, 6 times), *CCl*_*4*_ rats with systemic toxicity induced by CCl_4_ injection (1 mL/kg b.w., ip, 6 times), *CCl*_*4*_*-ASE* rats with systemic toxicity after their oral treatment with ASE (7.5 g/kg b.w.) for 10 days. *ASE*, normal rats were administered only ASE (7.5 g/kg b.w.) for 10 days. Different letters refer to the significance at *P* < 0.05; CCl_4_-ASE group was compared with the CCl_4_ group, while V and ASE groups were individually compared with the C group.

The results showed that CCl_4_ injection can also influence spleen oxidative stress and cause a massive elevation in ROS (292.700%) and NO (182.978%) levels, which led to an excessive generation of lipid peroxide (TBARS, 395.516%), compared to that of the C group (Fig. 5A,B). Likewise, there was a significant (*P* < 0.05) elevation in MPO activity (165.852%) compared to the control group (Fig. 5D). In contrast, there was a significant (*P* < 0.05) fall in the spleen antioxidant content, GPX (77.092%), GSH (75.735%), and SOD (50.388%), compared to the control group (Fig. 5C,D). Therefore, the TAC (63.063%) of spleen tissue was significantly (*P* < 0.05) diminished in the CCl_4_-injected rats, compared to the C group (Fig. 5A). Otherwise, the treatment of the CCl_4_-injected rats with ASE (CCl_4_-ASE group) enormously decreased the oxidative stress examined parameters, including ROS, NO, TBARS, and MPO level by 37.638%, 50.024%, 68.477%, 38.071%, sequentially, compared to those of the CCl_4_ group (Fig. 5A,B,D). Moreover, ASE enhanced the spleen antioxidant status by a significant (*P* < 0.05) elevation in the activity of GPX and SOD, and the level of GSH by 323.942%, 124.237%, and 126.792%, compared to those in the CCl_4_ group (Fig. 5C,D). In addition, the TAC of the spleen tissue was augmented by 195.244% more than the CCl_4_ group (Fig. 5A).

On the other hand, the spleen of rats that were injected with olive oil only for 3 weeks (V group) showed a slight increase in the levels of ROS (10.537%), TAC (0.677%), and TBARS (16.592%), compared to the C group (Fig. 5A,B). Similarly, there was a slight change in the activities of SOD (5.972%) and MPO (36.980%) (Fig. 5D). However, the level of NO was significantly (*P* < 0.05) increased by 36.504%. In contrast, the activity of GPX and the level of GSH were significantly (*P* < 0.05) reduced in the spleen of rats in this group by 15.281% and 25.714%, respectively, relative to the control rats (Fig. 5B,C). Further, the administration of ASE alone for 10 days (ASE group) showed a non-significant difference in the levels of splenic ROS (0.692%), TBARS (0.605%), NO (0.426%), and GSH (0.186%), compared to the C group. Likewise, the activities of MPO (5.071%), GPX (2.398%), and SOD (7.924%) were slightly changed. Nevertheless, the level of TAC (64.865%) was significantly (*P* < 0.05) increased in the spleen tissue after ASE intake, compared to that in the C group.

### The alleviating effect of ASE on CCl_4_-induced systemic necroinflammation

**Figure 6:**
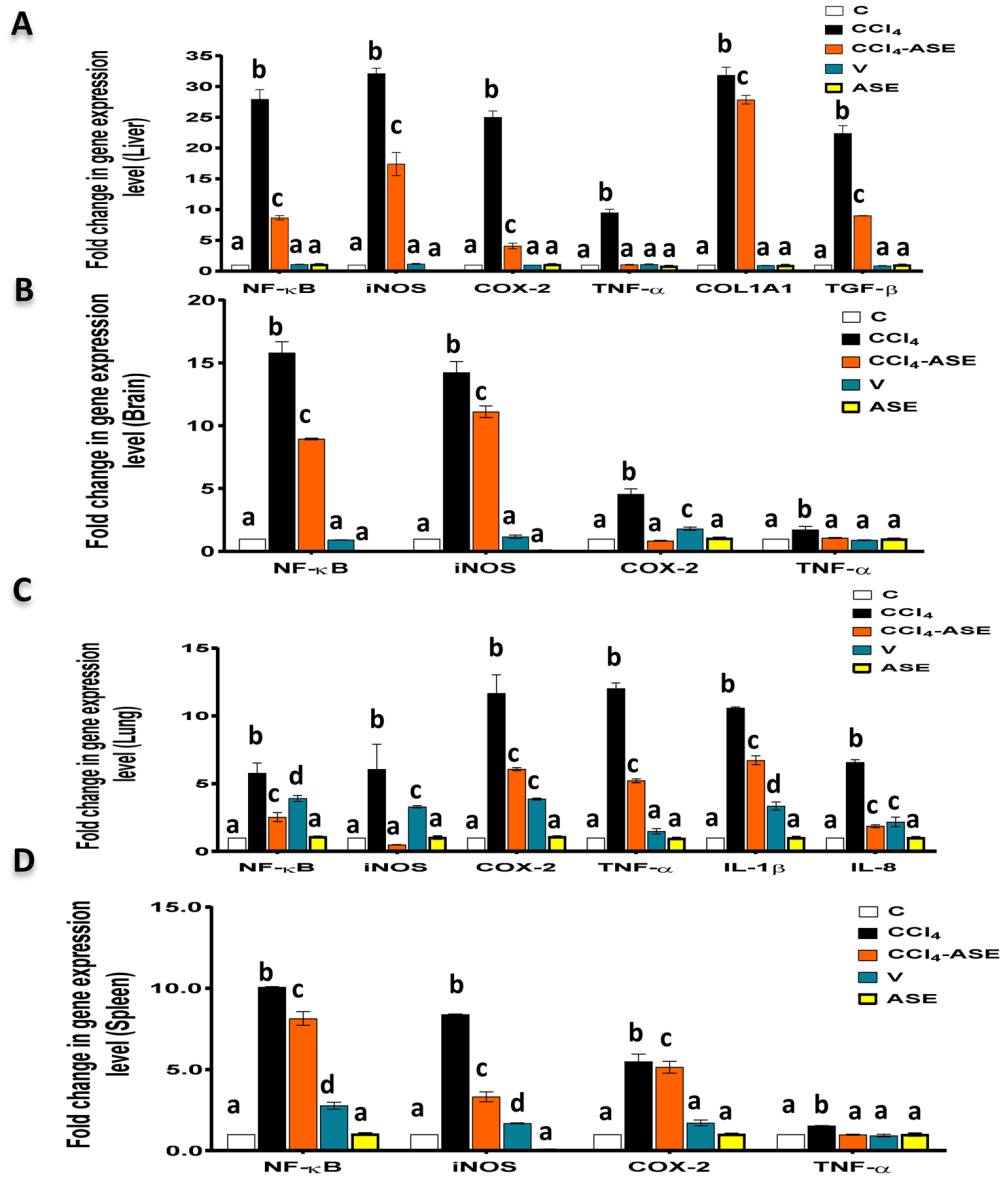
Ameliorating effect of ASE on the CCl_4_-induced systemic necroinflammation. (**A**–**D**) fold change in the gene expression of the pro-inflammatory mediators [nuclear factor-kappa (NF-κ)B, inducible nitric oxide synthase (iNOS), cyclooxygenase (COX)-2, and tumor necrosis factor (TNF)-α] in the liver, brain, lung, and spleen tissues, respectively. (**A**) and (**D**) also show the fold change in the gene expression of the hepatic profibrotic mediators [collagen type I alpha one chain (COL1A1) and transforming growth factor (TGF)-β] and lung-specific pro-inflammatory cytokines [interleukin (IL)-1β and IL-8], respectively. Results are expressed as mean ± S.E of 7 animals. *C* control untreated rats, *V* olive oil (vehicle of CCl_4_)-administered rats (0.5 ml/kg b.w., ip, 6 times), *CCl*_*4*_ rats with systemic toxicity induced by CCl_4_ injection (1 mL/kg b.w., ip, 6 times), *CCl*_*4*_*-ASE* rats with systemic toxicity after their oral treatment with ASE (7.5 g/kg b.w.) for 10 days. *ASE* normal rats were administered only ASE (7.5 g/kg b.w.) for 10 days. Different letters refer to the significance at *P* < 0.05; CCl_4_-ASE group was compared with the CCl_4_ group, while V and ASE groups were individually compared with the C group.

Figure 6 demonstrates the toxic effect of CCl_4_ on the studied organs by upregulating the gene expression of some pro-inflammatory and pro-fibrotic cytokines. Hence, the fold expression of NF-κB, iNOS, COX-2, and TNF-α, was highly upregulated after CCl_4_ injection, compared to the control group. These elevations were observed in the liver (2691.194%, 3110.867%, 2400.977%, and 847.982%, respectively), the brain (1479.924%, 1322.286%, 355.513%, and 71.140%, respectively), the lung (478.041%, 455.108%, 1066.674%, and 1101.253%, respectively), and the spleen (907.976%, 737.934%, 449.203%, and 52.784%, respectively). Furthermore, the fold expression of the hepatic pro-fibrotic cytokines (collagen type I alpha one chain "COL1A1", transforming growth factor-β1 "TGF-β1") was massively increased after CCl_4_ injection by 3082.635% and 2138.017%, respectively, compared to those in the C group (Fig. 6A). The results also detected a significant (*P* < 0.05) elevation in the fold expression of interleukin (IL)-1β (957.250%) and IL-8 (557.650%) in the lung tissue of rats in the CCl_4_ group, compared to those in the C group (Fig. 6C).

As shown in the graphs of Fig. 6, the treatment with ASE (ASE-CCl_4_ group) significantly (*P* < 0.05) reduced the fold expression of the pro-inflammatory cytokines (NF-κB, iNOS, COX-2, and TNF-α) compared to the CCl_4_ group. The percentages of this depletion in the liver and brain tissues were 68.969%, 45.839%, 83.713%, and 89.234%, respectively, and 43.415%, 21.889%, 81.595%, and 37.826%, respectively. Moreover, these percentages in the lung and spleen tissues were 56.183%, 91.091%, 47.988%, and 56.609%, respectively, and 41.020%, 19.242%, 6.393%, and 35.778%, respectively. Also, ASE was capable of depleting the fold expression of IL-1β (36.460%) and IL-8 (71.668%) in lung tissue, as well as COL1A1 (12.500%) and TGF-β1 (59.812%) in liver tissue, compared to those in the CCl_4_ group.

The present study reported that the administration of olive oil only (V group) did not greatly influence the fold expression of the pro-inflammatory and profibrotic cytokines in the liver compared to those in the control group (Fig. 6A). Hence, the percentage changes of these mediators, including NF-κB, iNOS, COX-2, TNF-α, COL1A1, and TGF-β1,compared to those in the control group, were 7.250%, 15.535%, 0.686%, 8.699%, 7.412, and 15.794%, respectively. Similarly, in the brain tissue, there was a small change in the fold expression of the studied inflammatory mediators (9.327%, 16.204%, and 10.658%, respectively), except for the COX-2, which showed a significant upregulation in its fold expression by 79.0641%, compared to that in the C group (Fig. 6B). While in the lung, the fold expression of the pro-inflammatory cytokines, comprising of NF-κB, iNOS, COX-2, TNF-α, IL-1β, and IL-8 were significantly (*P* < 0.05) upregulated compared to those in the C group (Fig. 6C). The percentage changes of these mediators were 290.183%, 229.548%, 286.581%, 48.410%, 234.688%, and 117.364%, respectively. Regarding the spleen tissue, there was a significant (*P* < 0.05) elevation in the fold expression of some pro-inflammatory mediators (NF-κB "177.005%", iNOS "68.012%"), and no changes in the others (COX-2 "71.528%", TNF-α "6.648%") after the injection with the olive oil, compared to those in the C group (Fig. 6D).

Further, the intake of ASE alone (ASE group) demonstrated a non-significant change in the fold expression of NF-κB, iNOS, COX-2, and TNF-α in the liver (6.735%, 98.537%, 6.673%, and 20.418%, respectively) and brain (99.846%, 94.732%, 4.284%, 1.494%, respectively) as shown in Fig. 6A,B. The same results were obtained with lung (7.488%, 2.495%, 7.346%, and 4.403%, respectively) and spleen (2.799%, 95.119%, 1.866%, and 0.578%, respectively) tissues (Fig. 6C,D). Besides, the fold expression of COL1A1 (9.421%) and TGF-β1 (2.564%) in the liver tissue (Fig. 6A) and IL-1β (1.072%) and IL-8 (1.386%) in the lung tissue (Fig. 6C) was slightly changed compared to those in the C group.

### Comparison of the therapeutic efficacy of ASE in the four studied organs

Supplementary Fig. 1 represents the heatmap plot, which clusters the oxidative stress and necroinflammation tested parameters that were observed after the treatment with ASE (CCl_4_-ASE) in the different studied organs. The color in the heatmap elucidates the quantity (% increase relative to the CCl_4_ group) of these different studied parameters, the higher the quantity, the darker the color. Figure 1A shows two clusters, one for the liver and spleen, and the other for the lung and brain. This figure illustrates the high capability of ASE in elevating the level of TAC and the activity of GPX and SOD in the liver and spleen, more than the lung and brain. However, it increased the GSH level in the lung more than in the other organs. While the heatmap in Supplementary Fig. 1B demonstrates two clusters, the liver with the lung and the brain with the spleen. The figure shows the ability of ASE to decrease the levels of ROS, NO, TBARS, NF-κB, iNOS, and TNF-α, and the activity of MPO in the liver and lung more than the brain and the spleen. Therefore, these heatmaps revealed that the liver was the most influenced tissue, and the therapeutic values of ASE and the brain were the lowest.

### Histopathological results

**Figure 7:**
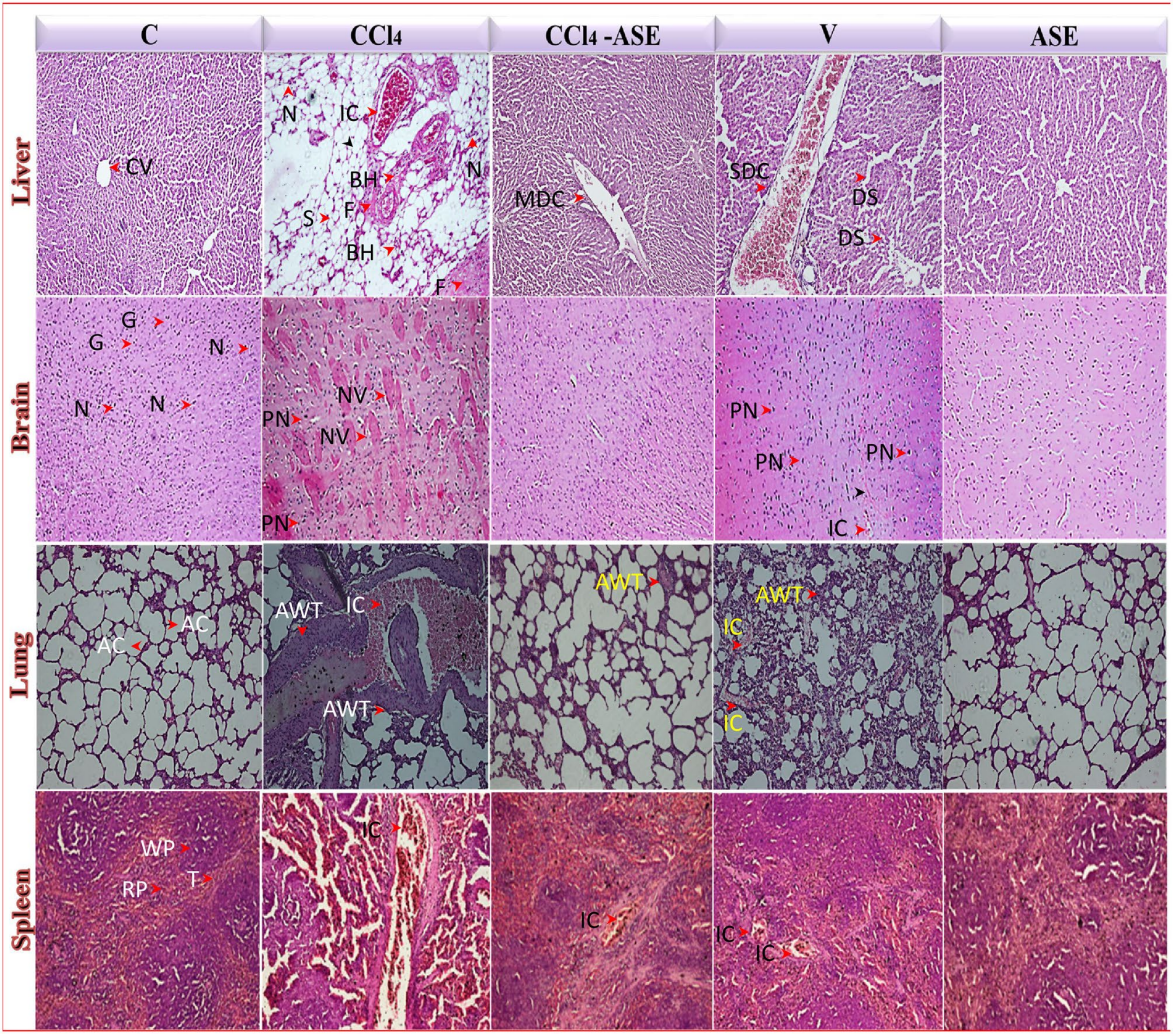
Photomicrographs (H&E stain, 20 ×) for liver, brain, lung, and spleen sections of the different studied groups revealed the ameliorating effects of ASE against the systemic toxicity induced by CCl_4_ injection. *AC* alveolar cells, *AWT*: alveolar wall thickness, *BH* binucleated hepatocytes, *CV* central vein, *DS* dilation of sinusoidal cells, *F* fibrous bands, *G* glial cells, *IC* inflammatory cells, *MDC* mild dilation of central vein, *N* neurons, *NV* neurons with the vacuole, *PN* neurons with pyknotic nuclei, *RP* red pulp, *SDC* severe dilation of the central vein, *S* steatosis, *T* trabecula, *WP* white pulp.

The microscopic examination of the processed formalin-fixed sections of the studied organs revealed the systemic toxicity of CCl_4_ and the ameliorating impact of ASE on the different tested organs (Fig. 7). The control sections of the liver revealed the hepatic central vein (CV) with normal lobular architecture and radiating liver cell cords. The CCl_4_ injection showed severe hepatotoxicity, as evidenced by severe steatohepatitis with fibrous bands. In addition, the features of necroinflammation such as congested blood vessels with infiltration of mononuclear leucocytes and necrotic and binucleated hepatocytes were observed. The treatment with ASE (CCl_4_-ASE) restored the normal morphology of the liver with only a mild dilation of the CV. Injection with olive oil only (V group) showed severe dilation of the CV with infiltration of mononuclear leucocytes, as well as mild sinusoidal cell activation. However, no pathological changes were detected in the liver of rats that were administered ASE only for 10 days (ASE group).

Regarding the brain, the control tissues showed normal architecture with normal glial cells and neurons. This normal morphology was changed after CCl_4_ injection to severe damage with severe degeneration of neurons that was characterized by the appearance of pyknotic nuclei and vacuoles in the neuronal cells. Administration of ASE restored the architecture of the brain and relieved the CCl_4_ toxicity. Injection of rats with olive oil (V group) caused mild degeneration of the neurons, which appeared with pyknotic nuclei and vacuoles in addition to the accumulation of the inflammatory cells in their vicinity. All these features reflected the induction of necroinflammation in the brain tissue of rats in the V group, while the oral administration of ASE alone (ASE group) didn’t affect the normal morphology of the brain.

The lung tissue in control rats had the normal appearance of the alveolar sacs and alveolar walls. Massive disruption was observed in its morphology after injection with CCl_4_, which is characterized by inflammatory cells influx with alveolar wall thickness and alveolar epithelium damage. Great amelioration in the lung morphology was observed following the administration of ASE (CCl_4_-ASE) compared to those in the CCl_4_ group. Hence, the lung tissue seemed normal with only mild thickness in the alveolar septae. In contrast, thickness in the alveolar septae with infiltration of the inflammatory cells and narrowing of the alveolar air spaces have appeared in the lung tissue sections of the animals in the V group. While no abnormal features were observed in the morphology of lung tissue in the rats of the ASE group.

Regarding the spleen tissue, it appeared normal with well-defined red and white pulp regions in the control rats. Severe disturbance in these regions with extreme infiltration of inflammatory cells was detected following the injection with CCl_4_. This damage was relieved after the intake of ASE (CCl_4_-ASE), except for the slight influx of inflammatory cells. Moderate disorganized white pulp compartment with unclear regions and mild recruitment of inflammatory cells was observed in the spleen tissues of the rats in the V group. However, the spleen tissues of animals in the ASE group showed normal architecture and well-distinct white and red pulp compartments like those in the control group.

## Discussion

AS gum has been well-known since ancient times and consists mainly of complex sugars such as galactose, rhamnose, arabinose, and glucuronic acid with about 25% proteins. In addition, the current study detected the presence of some amounts of phenolic compounds such as gallic, ellagic, benzoic, and O-coumaric acids (Fig. 1A). The presence of these compounds explains the current outcomes of the antioxidant activities of AS gum in vitro through scavenging the ABTS, NO, and lipid peroxide radicals. Hence, the antioxidant activities of phenolic acids and reducing sugars have been extensively reported in previous research^2,16,25,26^.

AS gum is used in the traditional medicine of the Arabs to improve renal function in patients with chronic kidney disease^27^. The present study evaluated its effect on the CCl_4_-induced systemic toxicity in rats. This highly toxic chemical causes metabolomics variations in the mammalian liver, which have been widely investigated, but it has been reported less frequently in other organs^12,28,29^. To examine the systemic toxicity induced by CCl_4_, we investigated the alterations in oxidative stress, inflammation, and necrosis markers in rat liver, lung, brain, and spleen. CCl_4_ is a common hepatic toxin that induces oxidative stress, necroptosis, fibrosis, and inflammation in rats^2,29^. This can be due to the liver’s metabolism, which is caused by CYP2E1. This catabolic process results in the formation of CCl_3_*, which is converted to the additional reactive trichloromethyl peroxyl radical (CCl_3_OO*), following its combination with oxygen^2,30^. The formation of these radicals will augment the ROS level in the liver and induce a disruption of its redox state and in turn, oxidative stress, leading to hepatic damage. The present study confirmed this damage in the liver after CCl_4_ exposure through a significant (*P* < 0.05) depletion in the ALT activity and albumin level, relative to the C group. The previous studies reported also the ability of CCl_4_ to induce metabolomic changes in rat kidney, lung, and spleen^12,18^. In line with this study, the present work provided fundamental data on CCl_4_ toxicity to various rat organs. Hence, injection with CCl_4_ significantly (*P* < 0.05) induced disturbances in the oxidative stress indices and necroinflammation in the rat liver, brain, lung, and spleen tissues. This may be due to the free radical metabolites of CCl_4_ that induce oxidative stress and trigger the production of inflammatory mediators in the liver, leading to an inappropriate inflammatory response and alteration in the functions of other organs^12,28,29^. The present study observed a substantially high level of ROS in the brain, lung, and spleen tissues in addition to the liver tissue. Concomitant to this, the TAC, non-enzymatic (GSH), and enzymatic (GPX and SOD) antioxidants were dramatically dropped compared to those in the C group in all the studied organs after CCl_4_ injection. This probably occurred due to the high cellular content of the free radicals, including the NO radicals, in these tested organs that affect the cellular macromolecules, particularly the membrane lipids, causing lipid peroxidation (high TBARS level)^2,11^. Peroxidation of lipids led to further formation and accumulation of ROS in the studied rat organs and resulted in the exhaustion of the cellular antioxidants such as GSH, GPX, and SOD^5,31^. The elevation in the activity of MPO after CCl_4_ injection magnified the oxidative stress condition due to its vital role in the production of hypochlorous acid (HOCl) in the neutrophils, which interacted with and consumed the GSH. This reaction occurred in the presence of H_2_O_2_ and halide ions and served to increase lipid peroxidation^32–34^, resulting in severe depletion of the cellular redox environment (TAC) in all of the organs studied (Figs. 2, 3, 4 and 5). All these outcomes are in agreement with the previous studies of Shah et al., Ali et al., and Habashy et al.^3,22,18^.

The administration of ASE for 10 days to the rats with systemic toxicity alleviated the CCl_4_-induced organ damage. This was clearly noticed from the significant (*P* < 0.05) decrease in the levels of NO and TBARS and the activity of MPO, along with the significant elevation in the GSH, GPX, and SOD, compared to those in the CCl_4_ group. Subsequently, the balance between the ROS level and TAC of each organ was about the achievement, indicating a reduction in oxidative stress. These results were in accordance with the previous study of Amanullah^35^. The in vivo antioxidant influence of ASE was in line with our in vitro results that confirmed the scavenging ability of ASE against ABTS, NO, and lipid peroxides (Fig. 1B). The ameliorating role of ASE could be linked to its composition, comprising phenolic acids, sugars, amino acids, and minerals. Hence, the free radical scavenging and antioxidant enhancement capabilities of gallic, ellagic, benzoic, and O-coumaric acids were recorded previously^36^. The AS gum mainly contains (97%) sugars, including L-arabinose, D-galactose, rhamnose, and glucuronic acid, along with others^37^ that have reducing power and potent antioxidant activities^38^. AS gum also encompasses different types of amino acids such as histidine, tyrosine, and lysine, which are generally considered as antioxidant compounds^39^. In addition to this, copper and zinc are two essential minerals in the AS gum^40^ that play a vital role in the SOD antioxidant activity^41^. The current results (Supplementary Fig. 1, heatmap plots) revealed that the improving efficiency of ASE to the CCl_4_-induced oxidative stress in the liver was more than the other organs, although the liver is the most vulnerable human organ to all aspects of injury, especially those caused by chemical toxicity, due to its essential role in xenobiotic metabolism. This may be due to the ability of this organ to regenerate itself by replacing the dead cells with new ones. Liver regeneration is a counterbalancing response to liver injury and it is defined for some toxicants, including CCl_4_^42^.

Regarding the rats in the ASE group, all the studied oxidative stress parameters were similar to the control, except for the hepatic GPX activity and GSH level, as well as the splenic TAC level, which were all significantly upregulated. These results not only referred to the safety of ASE on the liver, brain, lung, and spleen, but also to this extract's antioxidant enhancement ability in the liver and spleen. This capability may be attributed to the ASE phenolics such as gallic and coumaric acids, which were reported previously to upregulate the hepatic gene expression of GPX and elevate the GSH level by increasing the level of E2-related factor (the antioxidant response element regulating transcription factor)^43^. The improvement in the splenic TAC may be associated with the ability of ASE to modulate other types of the complex antioxidant network in this organ than those investigated in this study.

The current study interestingly found that olive oil injections had a mild toxicity on the brain, lung, and spleen tissues. These findings were in line with the recent study of Kouka et al. and Habashy et al., who demonstrated the ability of olive oil administration to induce oxidative stress in certain tissues^44,18^. This may be influenced by the multiple intakes of this antioxidant-rich oil, which has led to the consumption of the cellular antioxidant defence biomolecules, implying a prooxidant effect, which is called antioxidative stress^45^. The differences in prooxidant activities of olive oil in different organs can be attributed to differences in the concentration, availability, time of persistence, and distribution of olive oil antioxidant molecules and their metabolites^44,46^. This assumes that these organs didn't make valuable adaptations after antioxidant ingestion, and the observed negative effect may be reversed if the olive oil was administered for a longer period^47^. Thus, the systemic toxicity in rats injected with CCl_4_ is associated with CCl_4_ itself, not the vehicle olive oil.

To further confirm the therapeutic potential of ASE on CCl_4_-induced systemic toxicity, we examined the level of inflammatory markers in the different studied tissues (Fig. 6). In addition, the fibrotic mediators were evaluated in the liver tissue. The present study found that the fold expression of NF-κB, iNOS, COX-2, and TNF-α was elevated in liver, brain, lung, and spleen tissues of the CCl_4_-injected rats. Moreover, the fold expression of IL-1β and IL-8 in the lung tissue, as well as the fibrotic mediators, including COL1A1 and TGF-β in the liver tissue, were significantly (*P* < 0.05) raised. The inflammatory process is the earliest healing and defence mechanism of tissue damage, and there is a serious concern about the commitment of the ROS in supporting this process. Hence, ROS can activate NF-κB and, in turn, it can result in upregulation of the gene expression of the relevant pro-inflammatory mediators such as iNOS, COX-2, and TNF-α^48^. Therefore, due to the increased level of ROS in the rat liver, brain, lung, and spleen after CCl_4_ injection, NF-κB with other pro-inflammatory cytokines were upregulated. Activation of the NF-κB pathway is interrelated to the steatohepatitis^49^ seen in histopathological photomicrographs after CCl_4_ injection (Fig. 7). The hepatic morphology of the rats in the CCl_4_ group also showed the presence of fibrous bands that may be linked to the upregulation of COL1A1 and TGF-β1 gene expression (Fig. 6). These two cytokines are secreted by activated hepatic stellate cells and are essential activators for hepatic fibrogenesis^50^. Further, IL-8, IL-1β, and TNF-α are the main responsible cytokines for the inflammatory cell infiltration into lung tissue. It has been reported that the key regulators of IL-8 expression are IL-1 and TNF-α^51^. TNF-α and IL-1β stimulate NO production by iNOS, which induces nitrosative stress due to the formation of the highly reactive peroxynitrite radical after NO interaction with superoxide radical. As a result, the lung tissue showed thickening in the airway wall (Fig. 7), which reflected the mucosal inflammation, increased mucus glands, muscle mass, and vessel area, as well as connective tissue deposition on the extracellular matrix^52^. The upregulation of COX-2 gene expression can also augment the CCl_4_-induced systemic damage by catalyzing the formation of prostaglandin H2 from arachidonic acid, along with the production of superoxide radicals^5^. Furthermore, TNF-α and IL-1β activate the cellular apoptosis and necrosis pathways by altering the balance of the receptor and the ligand^53^. On the other hand, the elevation of MPO activity also contributed to an increase in the systemic inflammatory response of CCl_4_ by activating neutrophils^33^. Therefore, the injection of CCl_4_ to rats induced systemic necroinflammation by recruitment of the inflammatory cells to the different studied organs (Fig. 7), as well as cell necrosis by upregulating the TNF-α fold expression. All of these results are in accordance with our previous recent studies^2,18^.

The administration of ASE (CCl_4_-ASE group) relieved the systemic inflammatory state induced by CCl_4_ by downregulating the gene expression of proinflammatory mediators, including the NF-κB, iNOS, COX-2, TNF-α in all the studied organs. Also, the gene expression of IL-1β and IL-8 in the lung tissue was significantly (*P* < 0.05) decreased, compared to those in the CCl_4_ group. Moreover, ASE ingestion significantly (*P* < 0.05) declined the hepatic profibrotic markers (COL1A1, TGF-β) compared to those in the CCl_4_ group. These results were in harmony with the histopathological data that showed an improvement in the morphology of the different studied organs (Fig. 7). The previously confirmed anti-inflammatory and antifibrotic effects of ASE^54,55^ may be related to its potent antioxidant activity and potential effect on reducing the main inflammatory inducer, ROS. All of these influences may be implied in the presence of the antioxidant polyphenols in ASE such as ellagic, gallic, benzoic, and O-coumaric acids^56^. These results were in line with the previous investigations that revealed the ability of AS gum to block or treat the toxic signs of some drugs such as aspirin, acetaminophen, and cisplatin. Additionally, it plays a possible prophylactic role against the toxicity of some chemicals, such as trichloroacetic acid and mercuric chloride^57–59^. The cluster heatmap plot (Supplementary Fig. 1) revealed that the anti-inflammatory potency of ASE was higher in the liver than in the other organs. This may be due to the regeneration ability of the liver that requires certain types of cytokines such as TNF-α, NF-κB, and other factors. Hence, TNF-α is one of the hepatocytes priming factors that enhances their proliferation in addition to its stabilization role to the NF-κB for hepatic regeneration^42^. Further, the administration of ASE alone for 10 days (ASE group) demonstrated non-significant changes in the studied proinflammatory and profibrotic mediators in all the investigated organs. Furthermore, the architecture and morphology of the studied organs are still well preserved.

On the other hand, the injection with olive oil only (V group) had no adverse effect on the liver but increased the gene expression of certain inflammatory cytokines in the brain, lung, and spleen tissues (Fig. 6). These adverse effects may be owed to the inability of these organs to make adaptations after the olive oil intake as discussed above^47^. These biochemical outcomes were in agreement with the histopathological findings that showed infiltration of the inflammatory cells in the brain, lung, and spleen, along with other pathological changes. However, slight changes were observed in the liver of rats in this group (Fig. 7).

## Conclusions

The current study revealed the toxic effect of CCl_4_ on rat liver, brain, lung, and spleen (systemic toxicity) and the therapeutic impact of ASE. The biochemical, molecular, and histopathological findings explained the mechanism of ASE in alleviating this toxicity. This was achieved by restoring the cellular redox state and downregulating the gene expression of NF-κB and its related ROS-mediated inflammatory cytokines (regulating ROS/NF-κB pathway) in the studied tissues. The phenolic compounds, sugars, and other valuable constituents of ASE are the main cause of this potency. Therefore, ASE can be a promising extract used for the treatment of the systemic toxicity induced by CCl_4_.

## Materials and methods

### Chemicals

Folin–Ciocalteau reagent, phenolic standards, ABTS, BHT, CCl_4_, 2′,7′-dihydro-dichlorofluorescein diacetate (DCFH-DA) probe, thiobarbituric acid (TBA), tetra methoxy propane (TMP), GSH, and O-dianisidine dihydrochloride (ODD) were purchased from Sigma-Aldrich (St. Louis, MO, USA). Gene JET RNA purification kit, cDNA synthesis kit, and SYBR green master mix 2X kit were provided by Thermo Fisher Scientific, USA. Forward and reverse primers were bought from Bioneer, Korea. ALT and AST, albumin, and protein kits were obtained from Biosystem, Spain. Other chemicals were received with a high grade.

### Animals

Fifty male Albino rats were obtained from MISR University for Science and Technology with pet welfare (assurance number: A5865-01). The rats were adapted under ordinary circumstances of about 30 °C temperature with a 12-h light–dark period for two weeks. Throughout this time, the rats had free access to tap water and regular nutrition as required. All experimental protocols and methodology were carried out following the Institutional Animal Care and Use Committee (IACUC) and approved by the Alexandria University Committee of Animal Care and Use. This study was carried out in compliance with the Animal Research: Reporting of In Vivo Experiments (ARRIVE) guidelines.

### Preparation of ASE

The AS gum (NCBI:txid138043) was provided from Egypt and handled to prepare the crude extract. It was crushed using an electric grinder (Telstar, Terrassa, Spain) to receive the powder; and 50 g of it was dissolved in 200 mL of distilled water and left for 24 h in a refrigerator (4 °C) to be dissolved completely. The solution was filtered to eliminate the impurities and then lyophilized to get the powdered extract (ASE), which was stored at − 20 °C until used.

### Phytochemical analysis of ASE

The total amounts of flavonoids and phenolics in ASE were assessed using spectrophotometric and chromatographic methods. Total phenolics were measured by Folin–Ciocalteau reagent and the calibration curve of gallic acid^60^. Total flavonoids were estimated using 5% sodium nitrite and 10% AlCl_3_. The developed yellow-colored product with ASE or the catechin standard was recorded at 510 nm^61^.

High-performance liquid chromatography (HPLC) using 12 different types of pure phenolic standards was used to detect the phenolics in ASE. Twenty microliters of ASE were distributed on the ZORBAX Eclipse Plus C18 column (100 mm × 4.6 mm Agilent Technologies, Palo Alto, CA, USA). The separation was accomplished at 284 nm using a mobile phase of methanol, acetonitrile, and 0.2% H_3_PO_4_ with a ternary linear elution gradient and a flow speed of 0.75 mL/min^62^.

### In vitro antioxidant activities of ASE

The antiradical (ABTS and NO radicals) activities and β-carotene-linoleate bleaching analysis of ASE were tested. The 50% inhibitory concentration (IC50) for each test was recognized by the GraphPad Instat software version 3.

The ABTS^+^ radical cation-decolorization assay was used to study the ability of ASE to neutralize ABTS^+^ radical to ABTS^63^. ASE or BHT (standard antioxidant) was mixed with ABTS^+^ radical (7 mM ABTS was incubated with 140 mM potassium persulphate for 16 h at 25 °C) in dark. Then the absorbance of the blue color was recorded at 734 nm to calculate the percentage inhibition of ABTS^+^ radical.

The NO scavenging ability of ASE was detected by measuring the generated bright-reddish-purple azo dye color from the Griess reaction at 490 nm. Sodium nitroprusside and Griess reagent (0.1% naphthylethylenediamine dihydrochloride, 2% phosphoric acid, and 1% sulfanilamide)^64^ were used in this reaction.

The anti-lipid peroxidation effect of ASE can be determined by examining the β-carotene-linoleate bleaching. The assay was performed using the emulsion of linoleic acid, β-carotene, and Tween-80^65^. The ability of ASE to scavenge the produced radicals from linoleic acid oxidation was indicated by the decrease in the rate of β-carotene bleaching. The absorbance of the β-carotene color was recorded at 490 nm immediately (a) and after 180 min (b). Then the value of the degradation rate (DR) of ASE, control (without extract), and BHT (standard) was calculated from the equation: [DR = ln (a/b) × (1/180)]. The ASE scavenging capability was determined as the percentage of inhibition from the formula: Antioxidant activity (%) = (DR_control_ − DR_ASE_/DR_control_) × 100.

### Evaluation of the systemic anti-toxicity effect of ASE

#### Experimental design

**Figure 8:**
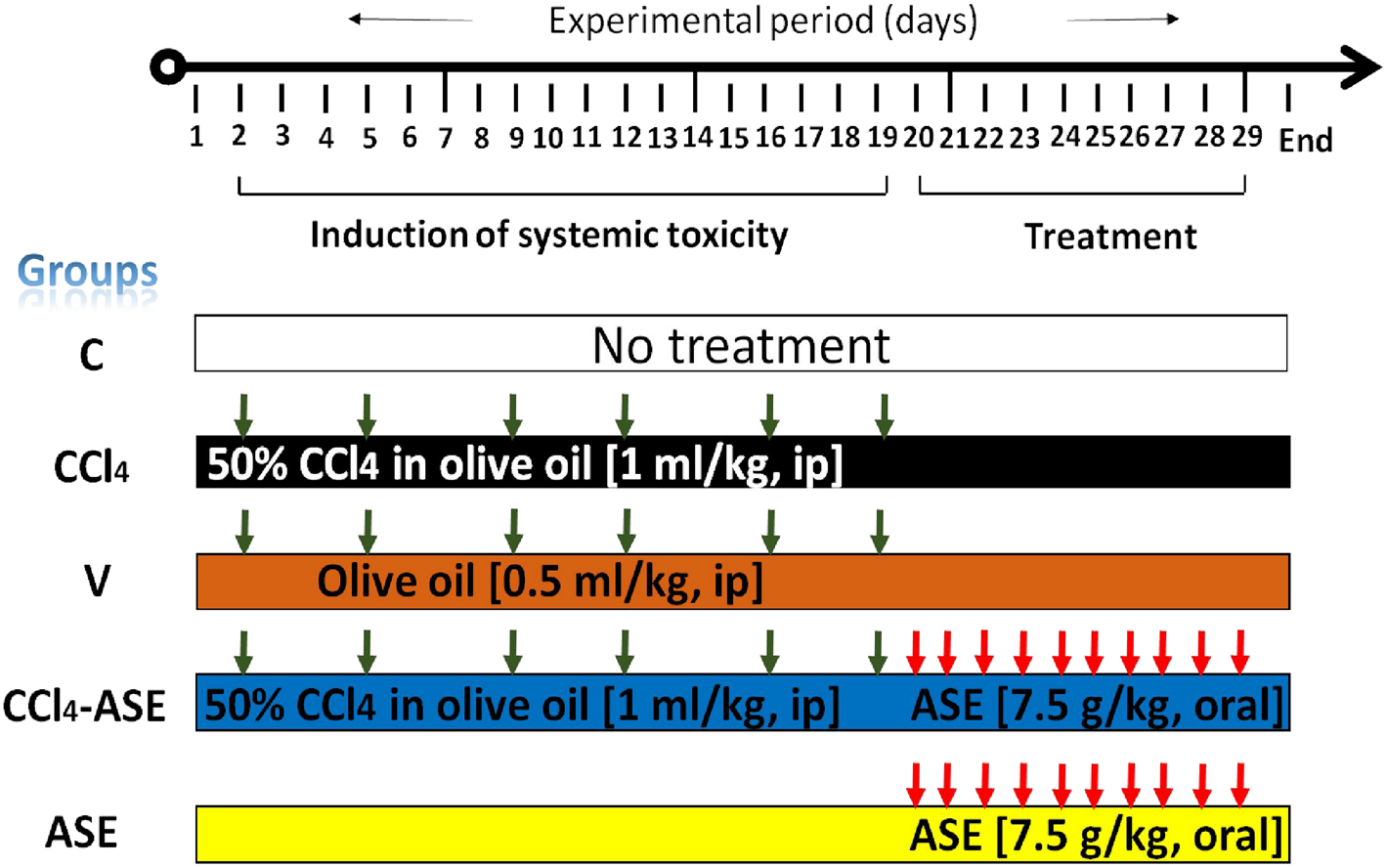
Experimental design with animal group treatments. Induction of systemic toxicity was performed by intraperitoneal (ip) injection of rats with CCl_4_ for 3 weeks (twice/week). Then rats were left without treatment for the period from day 20 to day 29. Animals in the CCl_4_-ASE group were orally gavaged with ASE for 10 days following induction of systemic toxicity. The study includes three control groups of animals, the untreated animals (C group, received no treatments in all the study period), animals in the V group that were injected for 3 weeks (twice/week) with the vehicle of CCl_4_ (olive oil, no treatment from day 20 to day 29), as well as those orally administered ASE for 10 days (ASE group, no treatment from day 0 to day 19).

Fifty male Albino rats (weighing 140–200 g, 6 weeks' age) were randomly divided into 5 groups (10 animals in each). The handling of animals in each laboratory group is presented in Fig. 8. Control rats received no treatment throughout the experimental period. The systemic toxicity was induced in rats by intraperitoneal (ip) injection with 50% CCl_4_ in olive oil (1 mL/kg b.w.) every Sunday and Wednesday for three weeks (then rats were left without treatment from day 20 to day 29)^66,67^. Similarly, rats in the V group were treated, but they were just given olive oil. The animals in the CCl_4_-ASE group were orally administered ASE (7.5 g/kg b.w., dissolved in water) by gavage, every day for ten days. While in the ASE group, rats were left without treatment from day 0 to day 19, then they were fed with ASE only (7.5 g/kg b.w., day 20–29). On day 30, rats were anesthetized by inhalation of isoflurane (2%) for 2 min and dissected immediately to obtain the blood by cardiac puncture and the tissues (liver, brain, lung, and spleen). The heparinized blood was centrifuged for 15 min at 1400×*g* to collect plasma for liver function parameters quantification. Liver, brain, lung, and spleen tissues were cleaned with cold saline (0.9% NaCl) and weighed. Then, small parts were fixed in 10% formalin for histopathological examination. The remaining tissues were stored at − 80 °C until utilized in the biochemical and molecular investigations.

#### Plasma investigation

Using the specific kits, the levels of liver function parameters (ALT, AST, total proteins, and albumin) in the separated plasma specimens of all the examined groups were measured colorimetrically.

#### Biochemical assessment of CCl_4_-induced systemic oxidative stress

The oxidative stress (cellular redox state disturbance) was determined in the studied organs by quantification of the intracellular ROS, TAC, lipid peroxidation, NO, MPO, and the enzymatic and non-enzymatic antioxidant markers. The organ homogenates were obtained by homogenizing one gram of each studied organ, separately in 10 mL of cold PBS. Then the cleared homogenates were collected after centrifugation for 30 min (1400×*g*, 4 °C) for the analyses^2^.

The concentration of ROS was determined by mixing an equal volume of the diluted homogenate (dilution with PBS, twofold) with 5 μM DCFH-DA fluorescent probe (diluted 1000-fold with 10 µM dimethyl sulfoxide). The amount of the fluorescence was recorded at 485 nm (excitation) and 520 nm (emission) after keeping the reaction mixture in the dark at 37 °C for 5 min. Then the ROS level in each studied organ was calculated using the H_2_O_2_ calibration curve^68^.

The TAC of the tested organs was determined using the ABTS radical cation method by mixing 2 mL of the radical solution with 20 µL of each organ homogenate or standard (BHT) or PBS (control)^63^. The preparation of ABTS solution and the method proceeded as indicated above in the in vitro assay, to obtain the percentage of inhibition. Then the TAC of each studied organ was calculated as BHT equivalent/g tissue using the BHT standard curve.

The level of lipid peroxidation was measured colorimetrically using the TBA reactive substances (TBARS) assay using the standard curve of TMP^69^. NO was examined using Griess reaction as referred to the in vitro part^65^.

The activity of MPO as μmoL of H_2_O_2_/min was quantified spectrophotometrically using 16.7 mg% ODD and 1.2% H_2_O_2_^70^, while the activity of the enzymatic antioxidants (GPX and SOD) were examined by Rotruck method^71^ and pyrogallol autooxidation method^41^, respectively. The specific activities of these enzymes were calculated as IU/mg protein using the value of the protein content in each organ homogenate that was measured by the biuret method using the specific kit. Ellman's reagent (5,5′-dithio bis2-nitrobenzoic acid) was used to determine the non-enzymatic antioxidant (GSH) molecule level using the GSH calibration curve^72^.

#### Real-time quantitative reverse transcription-polymerase chain reaction (qRT-PCR)

The necroinflammation in all the studied tissues and the fibrotic markers in the liver tissue were assessed at the molecular level using the qRT-PCR technique. The NF-кB, iNOS, COX-2, and TNF-α were evaluated here as good markers of necroinflammation in all the examined tissues, and IL-1β and IL-8 (essential pulmonary pro-inflammatory cytokines) were assessed in lung tissue. In addition, COL1A1 and TGF-β1 (critical hepatic fibrosis cytokines) were tested in liver tissues.

Each studied tissue was homogenized in lysis buffer containing β-mercaptoethanol and then centrifuged (3200×*g* for 5 min) and the total RNA was extracted from the obtained supernatant, using Gene JET RNA Purification Kit and quantified. Then the cDNA Synthesis Kit was used to synthesize the cDNA. The levels of target genes expression were determined via real-time PCR using SYBR green master mix kit, specific primers (Table 1), and β-actin as a housekeeping gene. The comparative Ct method (number of threshold cycles at cross-point between amplification plot and threshold) was used to calculate the fold expression of the studied genes^2^.

**Table 1:** Forward and reverse primer sequences used in the qRT-PCR.

Primer name	Primer sequences
NF-кB	Forward 5′-TGCTAATGGTGGACCGCAA-3′
	Reverse: 5′-CACTGCTTCCCGAATGTCTGA-3′
iNOS	Forward: 5′-ACCATGGAGCATCCCAAGTA-3′
	Reverse: 5′-CAGCGCATACCACTTCAGC-3′
COX-2	Forward: 5′-CCCATGTCAAAACCGTGGTG-3′
	Reverse: 5′-CTTGTCAGGAATCTCGGCGT-3′
TNF-α	Forward:5′-GCCCAGACCCTCACACTC-3′
	Reverse: 5′-CCACTCCAGCTGCTCCTCT-3′
COL1A1	Forward: 5′-CATGTTCAGCTTTGTGGACCT-3′
	Reverse: 5′-GCAGCTGACTTCAGGGATGT-3′
TGF-β1	Forward: 5′-TGCTAATGGTGGACCGCAA-3′
	Reverse: 5′-CACTGCTTCCCGAATGTCTGA-3′
IL-1β	Forward: 5′-GGGCCTCAAGGGGAAGAAGAATC-3′
	Reverse: 5′-ATGTCCCGACCATTGCTGTT-3′
IL-8	Forward: 5′-GAAGATAGATTGCACCGATG-3′
	Reverse: 5′-CATAGCCTCTCACACACATTTC-3′
β-Actin	Forward: 5′-AAGCAGGAGTATGACGAGTCCG-3′
	Reverse: 5′-GCCTTCATACATCTCAAGTTGG-3′

*NF-κB* nuclear factor-kappa B, *iNOS* inducible nitric oxide synthase, *COX-2* cyclooxygenase -2, *TNF-α* tumor necrosis factor-α, *COL1A1* collagen type I alpha one chain, *TGF-β1* transforming growth factor-β1, *IL* interleukin.

### Histopathological studies

The formalin-fixed tissue specimens were embedded in paraffin wax, then each sample was cut into small slices (5 µm thickness) and stained with hematoxylin and eosin following the standard histopathological examination protocol. Then, the phase-contrast microscope was used to visualize the pathological features of the examined tissues in all the studied groups, and high-resolution images (20 × magnification) were captured^2,72^.

### Statistical analysis

The results (symmetric with skewness values from 0 to 0.941) of the present study were expressed as mean ± S.E of seven rats. The parametric analysis, one-way analysis of variance (ANOVA) using Duncan’s test, was used to determine the difference between the mean values in all the studied groups. The analysis was done by SPSS software version 16 and the significance was considered at *P*-value < 0.05. The statistical significance between the studied variables was illustrated using different letters (a, b, c,…). The GraphPad Instate software version 3 was used to calculate the IC50 values for the in vitro antioxidant analyses.

## Supplementary Information

Supplementary Legends.

Supplementary Figures.
