# Supplementary material for: Acacia senegal gum attenuates systemic toxicity in CCl4-intoxicated rats via regulation of the ROS/NF-κB signaling pathway
Source: Sci Rep. 2021 Oct 13;11:20316. doi: 10.1038/s41598-021-99953-y (PMC8514504; doi:10.1038/s41598-021-99953-y)
Supplement: Supplementary file 1 — Supplementary Legends. [file 41598_2021_99953_MOESM1_ESM.pdf]

Supplementary Figure legend

Supplementary Fig. 1: Evaluation of the ASE therapeutic influence in the studied organs using heatmap plots. The heatmaps clustered the studied oxidative stress and necroinflammation parameters in the liver, brain, lung, and spleen tissues of the rats in the CCl_4-ASE group. Results are revealed as percentage change values in comparison to the CCl_4 group. **(A)** Heatmap distribution of the up-regulated parameters in the CCl_4-ASE group relative to the CCl_4 group. **(B)** Heatmap distribution of the down-regulated parameters in the CCl_4-ASE group relative to the CCl_4 group. The dark orange color refers to the higher percentage increase values and the light color indicates the lower ones.***COX-2***,cyclooxygenase-2; ***GPX***, glutathione peroxidase; ***GSH***, reduced glutathione; ***iNOS***,inducible nitric oxide synthase; ***MPO***, myeloperoxidase; ***NF-κB***, nuclear factor-kappa B; ***NO***, nitric oxide; ***ROS***, reactive oxygen species; ***SOD***, superoxide dismutase; ***TAC***, total antioxidant capacity; ***TBARS***, thiobarbituric acid reactive substances; ***TNF-α***, tumor necrosis factor-α**.**
