# Supplementary figures and images for: Acacia senegal gum attenuates systemic toxicity in CCl4-intoxicated rats via regulation of the ROS/NF-κB signaling pathway
Source: Sci Rep. 2021 Oct 13;11:20316. doi: 10.1038/s41598-021-99953-y (PMC8514504; doi:10.1038/s41598-021-99953-y)

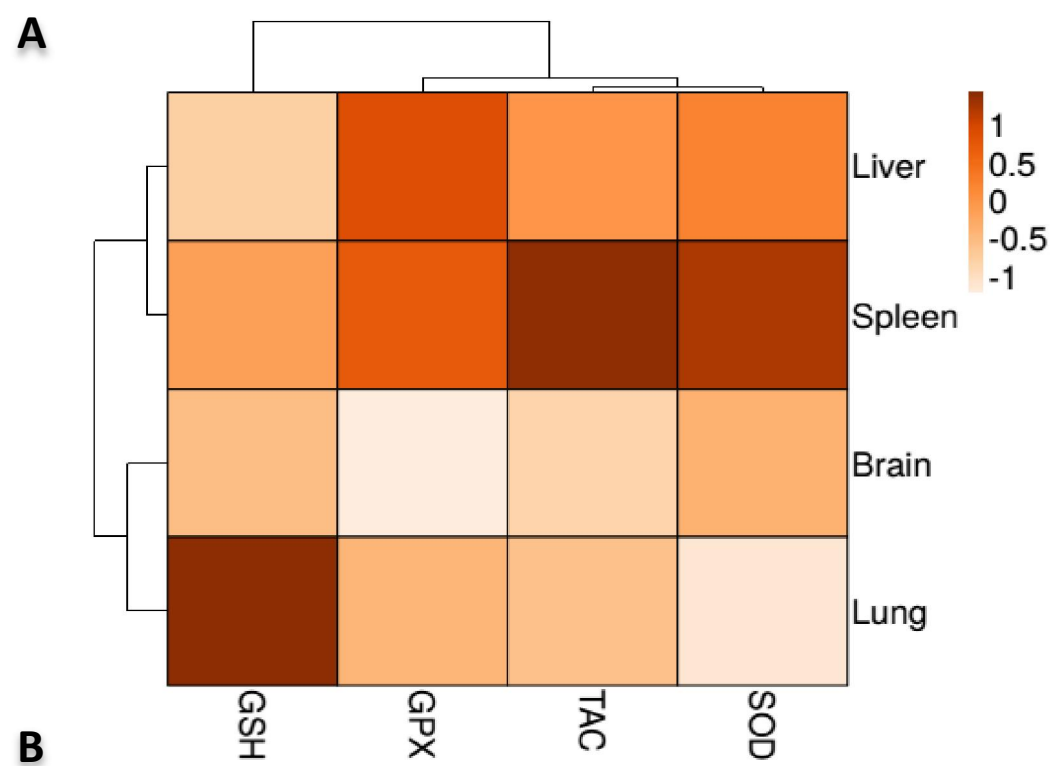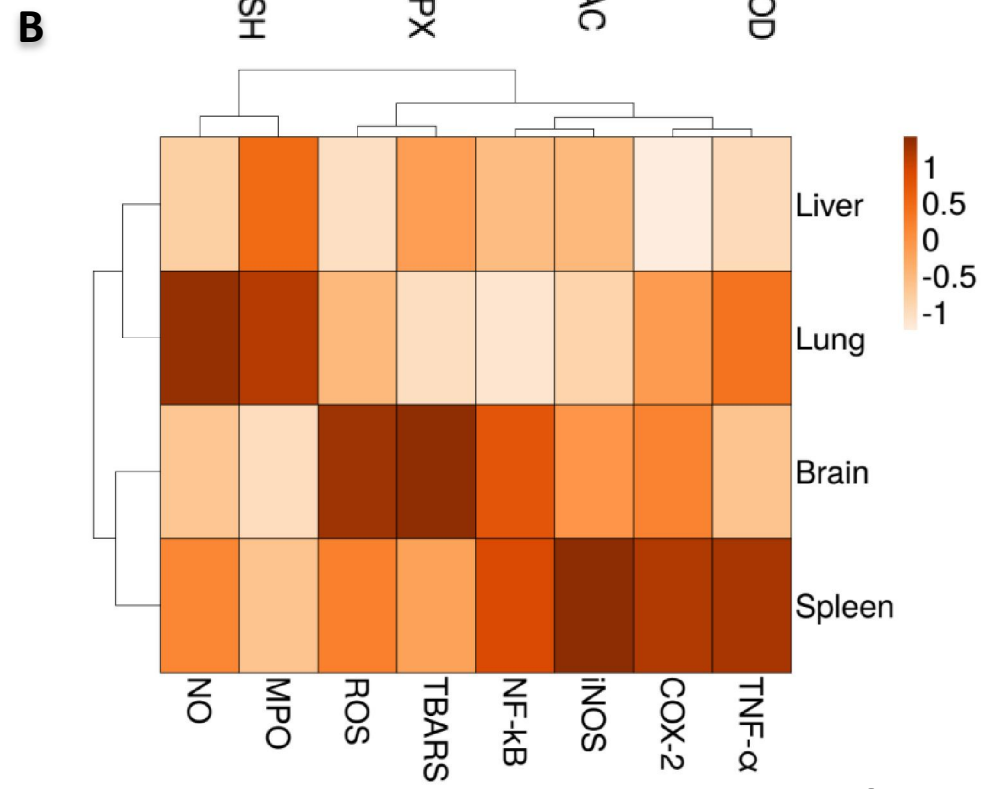

**Supplementary Figure 1**

Supplement: Supplementary file 2 — Supplementary Figures. [file 41598_2021_99953_MOESM2_ESM.pdf]
